# Association between socioeconomic status and cerebral palsy

**DOI:** 10.1371/journal.pone.0191724

**Published:** 2018-01-24

**Authors:** Sung-Hui Tseng, Jiun-Yih Lee, Yi-Lin Chou, Mei-Ling Sheu, Yuan-Wen Lee

**Affiliations:** 1 Department of Physical Medicine and Rehabilitation, Taipei Medical University Hospital, Taipei, Taiwan; 2 Department of Physical Medicine and Rehabilitation, School of Medicine, College of Medicine, Taipei Medical University, Taipei, Taiwan; 3 Quality Manage Center, Shin Kong Wu Ho-Su Memorial Hospital, Taipei, Taiwan; 4 Department of Physical Medicine and Rehabilitation, Shin Kong Wu Ho-Su Memorial Hospital, Taipei, Taiwan; 5 School of Health Care Administration, Taipei Medical University, Taipei, Taiwan; 6 Department of Anesthesiology, School of Medicine, College of Medicine, Taipei Medical University, Taipei, Taiwan; 7 Department of Anesthesiology, Taipei Medical University Hospital, Taipei, Taiwan; Boston Children's Hospital / Harvard Medical School, UNITED STATES

## Abstract

**Background:**

The present study investigated the annual prevalence of cerebral palsy (CP) among children aged <7 years in Taiwan and the association between socioeconomic status and CP prevalence.

**Methods:**

Data from the Taiwan National Health Insurance Research Database for the 2002−2008 period were used in this population-based study. Severe and total CP were defined according to catastrophic illness certificate and medical claim records, respectively. The annual CP prevalence was calculated as the number of children with CP among all children aged <7 years.

**Results:**

From 2002 to 2008, the annual prevalence of total and severe CP ranged from 1.9 to 2.8 and from 1.1 to 1.4 per 1000 children, respectively. Boys were 30% more likely to have CP than girls [adjusted relative risk (RR) and 95% confidence interval (CI) ranged from 1.3 (1.2−1.4) to 1.4 (1.2−1.5)]. Low family income was associated with a higher CP prevalence [adjusted RR (95% CI) ranged from 5.1 (4.2−6.2) to 6.4 (5.4−7.6)]. The prevalence of CP in rural area was higher than that in urban or suburban areas. The mortality rate of severe CP ranged from 12.2−22.7 per 1000 children within the 7 years study period.

**Conclusions:**

The prevalence of CP in Taiwan is similar to that in Western countries. A higher prevalence of CP is associated with male sex, low income, and rural residential location. Our findings provide insights into CP epidemiology among the Chinese population.

## Introduction

Cerebral palsy (CP) is the most common cause of childhood physical disability. The disabling conditions associated with CP impose considerable demands on the health, education, and social services of society. Therefore, it is imperative to obtain accurate and up-to-date estimates of the prevalence and associated risk factors of CP to project the burden of this condition and provide appropriate health resource allocations and effective preventive measures.

The worldwide prevalence of CP has been reported to be 2.1 per 1000 live births [1]. However, CP appeared to be more prevalent in low- or middle-income countries than in high-income countries [1–9]. CP prevalence was about 1.8 to 2.3 cases per 1000 children in Europe, Australia, and the USA, but the prevalence was 2.9 and 3.6 per 1000 children in Uganda and Egypt respectively [8,9]. Most of the reported overall prevalence rates have indicated no substantial changes for the birth-year periods of 1985 to 2010; however, a report based on the Surveillance of Cerebral Palsy in Europe demonstrated a decline in the prevalence of CP among children with birth body weight <1500 g [10]. Studies conducted in the United States have reported that Asian children had a lower CP prevalence than Caucasian [11,12]. Furthermore, surveys in China and Hong Kong have reported that the prevalence of CP was 1.3 to 1.6 per 1000 children, which is lower than that in Western countries [13–15]. However, it is unclear whether the lower prevalence of CP in Asian children can be attributed due to ethnic disparities.

Several nationwide, population-based CP registries from different continents have consistently reported a 6%−25% higher proportion of men with CP than women with CP [1,2,5]. Population-based and meta-analysis studies have also reported an association between low socioeconomic status (SES) and high CP prevalence [16–18]. In these studies, sex, ethnicity, and residential-area-based and individual-level SES influenced CP prevalence.

In Taiwan, the government-run single-payer National Health Insurance affords equity and high quality medical care to more than 99% of its 23 million citizens and foreign residents [19]. CP treatment is covered under Taiwan’s National Health Insurance. Therefore, the Taiwan National Health Insurance Research Database is an excellent tool with which to investigate the prevalence of CP in Taiwan. The present study investigated the annual prevalence of CP among children in Taiwan aged <7 years and the association between CP prevalence and SES by evaluating the data from the Taiwan Health Insurance Research Database recorded from 2002 to 2008. Our findings could provide insights into CP epidemiology among the Chinese population.

## Methods

### Data source

The Taiwan National Health Insurance Research Database was used in this study. Taiwan’s National Health Insurance is a mandatory and single-payer program that covers 99.9% of the approximately 23 million people residing in Taiwan [19,20]. The program covers all medically necessary services, including inpatient and outpatient services, dental care, and prescription drugs. The Taiwan National Health Insurance Research Database contains de-identified registration files and original claims data for reimbursement under the national health insurance program, and these data are provided to scientists for research purposes [21]. The demographic variables of the study population, including sex, low income, and residential location, were obtained from the registry for beneficiaries files. The registry for beneficiaries files had records of low income households, however, it did not contain detailed information on family income. Therefore, children were classified into low income status according to the records of low income households. We categorized the residential locations into urban, suburban, and rural areas according to the urbanization stratification published by the Taiwan National Health Research Institute [22]. The present study used all registration files and claims data of beneficiaries younger than 7 years between 2002 and 2008 for analysis. This study was approved by the Joint Institutional Review Board of Taipei Medical University (TMU-JIRB No. 210206046).

### Definition of severe CP

In the present study, children with CP were defined as those who aged 2 or older and had at least two claim records with ICD-9-CM codes 343.x within 1 year. Children with severe CP were those who had both definite CP diagnosis and moderate to severe physical or mental disability. In Taiwan, individuals diagnosed with severe and chronic diseases requiring extended treatment, such as CP, may apply for a catastrophic illness certificate. Patients with a catastrophic illness certificate are not required to make copayments for medical services. To obtain a catastrophic illness certificate, CP diagnosis must be confirmed by specialists [23]. In addition, the patient must be proven to have moderate to severe physical or mental disability by the designated hospitals [24]. Patients obtain this certificate after fulfilling the aforementioned provisions and verification by the National Health Insurance Administration. Therefore, we used the catastrophic illness registration file to define severe CP. Children having a catastrophic illness certificate with the International Classification of Disease, Ninth Revision, Clinical Modification (ICD-9-CM) diagnosis codes 343.x were defined as having severe CP. The number of total CP was defined as the number of children who fulfilled the above-mentioned criteria of CP or severe CP.

### Statistical analysis

The annual prevalence of CP was calculated by dividing the number of cases by the total number of children aged less than 7 years in the research database annually. Confidence intervals (CIs) were calculated by using the Poisson approximation to the binomial distribution. We used Poisson regression to analyze trends in prevalence over time by treating calendar year as a continuous variable. Multivariable Poisson regression was use to analyze the prevalence of CP after controlling for sex, low income, and residential area. Pearson χ^2^ tests were used to compare the prevalence of CP by sex, family income, and residential location. The baseline characteristics of the children, including the data on low family income and residential location, were obtained from the registry for beneficiary files. The date of death is included in the registry of catastrophic illness files. Accordingly, these death records were used to calculate the mortality rate of children with severe CP. A P-value of <0.05 was condisered statistically significant. All data were analyzed using SAS for Windows (version 9.3, SAS Institute, Cary, NC, USA).

## Results

The database covered nearly 99% of age-specific children in each specific year. The basic characteristic of study population are shown in Table 1. The number of children (age <7 y) in each year in the database indicated that Taiwan has a steadily declining fertility rate. The prevalence of total CP was 1.9 per 1000 children in 2002 (Table 2). Then the annual prevalence of total CP ranged from 2.5 to 2.8 per 1000 children and remained stable between 2003 and 2008 (*P* for trend = 0.843). For the period 2002 to 2008, the annual prevalence of severe CP ranged from 1.1 to 1.4 per 1000 children (Table 2). The prevalence rates of severe CP thus did not vary significantly throughout the 7-year study period (*P* for trend = 0.333). The proportion of boys with CP was consistently higher than that of girls with CP in both total and severe CP. Boys were 30% more likely to have CP than girls [relative risk and 95% confidence interval (CI) ranged from 1.3 (1.2−1.4) to 1.4 (1.2−1.5)] (Table 3).

**Table 1:** Basic characteristics of children aged 0−6 years in Taiwan between 2002 and 2008.

	2002 N (%)	2003 N (%)	2004 N (%)	2005 N (%)	2006 N (%)	2007 N (%)	2008 N (%)
Total number of children (age: 0–6 y)	2,080,296	2,028,332	1,899,332	1,838,358	1,768,354	1,687,999	1,599,331
Sex							
Boys	1,084,811 (52.2)	1,059,146 (52.2)	992,896 (52.3)	960,989 (52.3)	925,018 (52.3)	882,736 (52.3)	836,910 (52.3)
Girls	995,482 (47.9)	969,158 (47.8)	906,422 (47.7)	877,360 (47.7)	843,330 (47.7)	805,259 (47.7)	762,419 (47.7)
Unknown	3 (0.0)	28 (0.0)	14 (0.0)	9 (0.0)	6 (0.0)	4 (0.0)	2 (0.0)
Family income							
Low income	12,892 (0.6)	16,145 (0.8)	16,235 (0.9)	17,172 (0.9)	17,128 (1.0)	16,753 (1.0)	16,564 (1.0)
Middle or high income	2,067,404 (99.4)	2,012,187 (99.2)	1,883,097 (99.2)	1,821,186 (99.1)	1,751,226 (99.0)	1,671,246 (99.0)	1,582,767 (99.0)
Residential location							
Urban	860,692 (41.4)	852,900 (42.1)	795,749 (41.9)	771,691 (42.0)	741,775 (42.0)	711,880 (42.2)	679,115 (42.5)
Suburban	618,606 (29.7)	597,343 (29.5)	564,627 (29.7)	549,107 (29.9)	533,649 (30.2)	512,141 (30.3)	486,158 (30.4)
Rural	600,998 (28.9)	578,089 (28.5)	538,956 (28.4)	517,560 (28.2)	492,930 (27.9)	463,978 (27.5)	434,058 (27.1)

**Table 2:** Annual prevalence of total and severe CP in Taiwan children aged 0−6 years.

	2002	2003	2004	2005	2006	2007	2008
Total number of children (age: 0−6 y)	2,080,296	2,028,332	1,899,332	1,838,358	1,768,354	1,687,999	1,599,331
Total number of children (age: 0−6 y) with CP	4025	5157	5383	5187	4941	4647	4077
Total CP prevalence (95% CI) per 1000 children	1.9 (1.9−2.0)	2.5 (2.5−2.6)	2.8 (2.8−2.9)	2.8 (2.7−2.9)	2.8 (2.7−2.9)	2.8 (2.7−2.8)	2.5 (2.5−2.6)
Number of children (age: 0−6 y) with severe CP	2270	2681	2601	2432	2289	2068	1763
Severe CP prevalence (95% CI) per 1000 children	1.1 (1.0−1.1)	1.3 (1.3−1.4)	1.4 (1.3−1.4)	1.3 (1.3−1.4)	1.3 (1.2−1.3)	1.2 (1.2−1.3)	1.1 (1.1−1.2)

**Table 3:** Prevalence of total and severe CP according to sex.

	2002	2003	2004	2005	2006	2007	2008
Total number of boys with CP	2342	3028	3158	3050	2930	2739	2382
Total number of girls with CP	1683	2129	2225	2137	2011	1908	1695
Total CP prevalence (95% CI) per 1000 children							
Boys	2.2 (2.1−2.2)	2.9 (2.8−3.0)	3.2 (3.1−3.3)	3.2 (3.1−3.3)	3.2 (3.1−3.3)	3.1 (3.0−3.2)	2.8 (2.7−3.0)
Girls	1.7 (1.6−1.8)	2.2 (2.1−2.3)	2.5 (2.4−2.6)	2.4 (2.3−2.5)	2.4 (2.3−2.5)	2.4 (2.3−2.5)	2.2 (2.1−2.3)
Relative risk (95% CI) (boys/girls)	1.3 (1.2−1.4)	1.3 (1.2−1.4)	1.3 (1.2−1.4)	1.3 (1.2−1.4)	1.3 (1.3−1.4)	1.3 (1.2−1.4)	1.3 (1.2−1.4)
Number of boys with severe CP	1326	1581	1546	1452	1363	1217	1037
Number of girls with severe CP	944	1100	1055	980	926	851	726
Severe CP prevalence (95% CI) per 1000 children							
Boys	1.2 (1.2−1.3)	1.5 (1.4−1.6)	1.6 (1.5−1.6)	1.5 (1.4−1.6)	1.5 (1.4−1.6)	1.4 (1.3−1.5)	1.2 (1.2−1.3)
Girls	0.9 (0.9−1.0)	1.1 (1.1−1.2)	1.2 (1.1−1.2)	1.1 (1.0−1.2)	1.1 (1.0−1.2)	1.1 (1.0−1.1)	1.0 (0.9−1.0)
Relative risk (95% CI) (boys/girls)	1.3 (1.2−1.4)	1.3 (1.2−1.4)	1.3 (1.2−1.4)	1.4 (1.2−1.5)	1.3 (1.2−1.5)	1.3 (1.2−1.4)	1.3 (1.2−1.4)

We also investigated the association of CP prevalence with family income and residential status. Compared with children from middle- or high-income families, the children from low-income families were associated with a fivefold higher prevalence of total and severe CP [relative risk (95% CI) ranged from 4.9 (4.3−5.6) to 6.6 (5.5−7.8)] (Table 4). With regard to residential-location based SES, the prevalence of total and severe CP in rural areas was higher than that in urban or suburban areas (Table 5). On the multivariable Poisson regression model controlling for confounders, the boys, low income, and rural residential location remained associated with higher prevalence of severe CP between 2002 and 2008 (Table 6). The mortality rate of severe CP ranged from 12.2 to 22.7 per 1000 children within the 7 years study period (Table 7).

**Table 4:** Prevalence of total and severe CP according to family income status.

	2002	2003	2004	2005	2006	2007	2008
Total number of children with CP							
Low income	133	218	218	249	248	234	213
Middle or high income	3892	4939	5165	4938	4693	4413	3864
Total CP prevalence (95% CI) per 1000 children							
Low income	10.3 (8.7−12.2)	13.5 (11.8−15.4)	13.4 (11.8−15.3)	14.5 (12.8−16.4)	14.5 (12.8−16.4)	14.0 (12.3−15.9)	12.9 (11.2−14.7)
Middle or high income	1.9 (1.8−1.9)	2.5 (2.4−2.5)	2.7 (2.7−2.8)	2.7 (2.6−2.8)	2.7 (2.6−2.8)	2.6 (2.6−2.7)	2.4 (2.4−2.5)
Relative risk (95% CI) (low income/middle or high income)	5.5 (4.6−6.5)	5.5 (4.8−6.3)	4.9 (4.3−5.6)	5.3 (4.7−6.1)	5.4 (4.8−6.1)	5.3 (4.6−6.0)	5.3 (4.6−6.0)
Total number of children with severe CP							
Low income	77	134	126	129	121	103	101
Middle or high income	2193	2547	2475	2303	2168	1965	1662
Severe CP prevalence (95% CI) per 1000 children							
Low income	6.0 (4.8−7.5)	8.3 (7.0−9.8)	7.8 (6.5−9.2)	7.5 (6.3−8.9)	7.1 (5.9−8.4)	6.1 (5.1−7.5)	6.1 (5.0−7.4)
Middle or high income	1.1 (1.0−1.1)	1.3 (1.2−1.3)	1.3 (1.3−1.4)	1.3 (1.2−1.3)	1.2 (1.2−1.3)	1.2 (1.1−1.2)	1.1 (1.0−1.1)
Relative risk (95% CI) (low income/middle or high income)	5.6 (4.5−7.1)	6.6 (5.5−7.8)	5.9 (4.9−7.1)	5.9 (5.0−7.1)	5.7 (4.8−6.9)	5.2 (4.3−6.4)	5.8 (4.8−7.1)

**Table 5:** Prevalence of total and severe CP according to residential location.

	2002	2003	2004	2005	2006	2007	2008
Total number of children with CP							
Urban	1660	2109	2188	2162	2043	1950	1698
Suburban	1193	1511	1533	1506	1444	1339	1200
Rural	1172	1537	1662	1519	1454	1358	1179
Total CP prevalence (95% CI) per 1000 children							
Urban	1.9 (1.8−2.0)	2.5 (2.4−2.6)	2.7 (2.6−2.9)	2.8 (2.7−2.9)	2.8 (2.6−2.9)	2.7 (2.6−2.9)	2.5 (2.4−2.6)
Suburban	1.9 (1.8−2.0)	2.5 (2.4−2.7)	2.7 (2.6−2.9)	2.7 (2.6−2.9)	2.7 (2.6−2.8)	2.6 (2.5−2.8)	2.5 (2.3−2.6)
Rural	2.0 (1.8−2.1)	2.7 (2.5−2.8)	3.1 (2.9−3.2)	2.9 (2.8−3.1)	2.9 (2.8−3.1)	2.9 (2.8−3.1)	2.7 (2.6−2.9)
Total number of children with severe CP							
Urban	914	1044	980	960	925	847	722
Suburban	629	747	726	692	641	576	482
Rural	727	890	895	780	723	645	559
Severe CP prevalence (95% CI) per 1000 children							
Urban	1.1 (1.0−1.1)	1.2 (1.2−1.3)	1.2 (1.2−1.3)	1.2 (1.2−1.3)	1.2 (1.2−1.3)	1.2 (1.1−1.3)	1.1 (1.0−1.1)
Suburban	1.0 (0.9−1.1)	1.3 (1.2−1.3)	1.3 (1.2−1.4)	1.3 (1.2−1.4)	1.2 (1.1−1.3)	1.1 (1.0−1.2)	1.0 (0.9−1.1)
Rural	1.2 (1.1−1.3)	1.5 (1.4−1.6)	1.7 (1.6−1.8)	1.5 (1.4−1.6)	1.5 (1.4−1.6)	1.4 (1.3−1.5)	1.3 (1.2−1.4)

**Table 6:** Multivariate Poisson regression analysis of the relative risk of severe CP.

	2002	2003	2004	2005	2006	2007	2008
Variable	Relative risk (95% CI)						
Sex							
Girls	1.0	1.0	1.0	1.0	1.0	1.0	1.0
Boys	1.3 (1.2−1.4)	1.3 (1.2−1.4)	1.3 (1.2−1.5)	1.4 (1.3−1.5)	1.4 (1.2−1.5)	1.3 (1.2−1.4)	1.3 (1.2−1.4)
Family income							
Middle or high income	1.0	1.0	1.0	1.0	1.0	1.0	1.0
Low income	5.5 (4.4−6.9)	6.4 (5.4−7.6)	5.8 (4.8−6.9)	5.8 (4.8−6.9)	5.6 (4.6−6.7)	5.1 (4.2−6.2)	5.6 (4.6−6.9)
Residential location							
Urban	1.0	1.0	1.0	1.0	1.0	1.0	1.0
Suburban	1.0 (0.9−1.1)	1.0 (0.9−1.1)	1.1 (1.0−1.2)	1.0 (0.9−1.1)	1.0 (0.9−1.1)	1.0 (0.9−1.1)	1.0 (0.8−1.1)
Rural	1.1 (1.0−1.2)	1.2 (1.1−1.3)	1.3 (1.2−1.5)	1.2 (1.1−1.3)	1.1 (1.0−1.3)	1.1 (1.0−1.3)	1.2 (1.1−1.3)

**Table 7:** Annual mortality rate of severe CP in Taiwan children aged 0−6 years.

	2002	2003	2004	2005	2006	2007	2008
Number of deaths in children (age: 0−6 y) with severe CP	40	41	59	49	28	35	40
Mortality rate (95% CI) per 1000 children	17.6 (12.9−24.0)	15.3 (11.3−20.8)	22.7 (17.6−29.3)	20.1 (15.3−26.7)	12.2 (8.4−17.7)	16.9 (12.2−23.6)	22.7 (16.6−30.9)

## Discussion

This study was the first nationwide population-based analysis of the prevalence and associated socioeconomic risk factors of CP in children aged <7 years in Taiwan over a 7-year period. The prevalence of CP remained constant during the study period. The present results yielded a peak CP prevalence of 2.8 per 1000 children, which is similar to those reported in a previous meta-analysis for a specific age range [3] but higher than those of other surveys in the Chinese population. The prevalence of CP in China and Hong Kong was reported to be from 1.3 to 1.6 per 1000 children [13–15], which is lower than that in other developed countries. However, our present findings based on the entire population of Taiwan provide new evidence that Chinese children do not have a lower CP risk.

Our results revealed that sex influences the prevalence of CP; a higher prevalence of CP was discovered in boys than girls, which is consistent with previous studies [25, 26]. In addition, CP prevalence was higher among the children of low-income families, which is in accordance with previous findings [11,27]. In the United States and United Kingdom, socioeconomic deprivation is associated with an increased risk of having a child with CP. A socioeconomic gradient has been observed in preterm birth, low birthweight, and postnatal injury, which are also risk factors for CP [18,28]. Some mediators have been suggested to explain the relationship between socioeconomic gradient and CP prevalence, including maternal illness, infection, inadequate prenatal care, poor nutrition, alcoholism, and smoking [11,28–31]. Strategies to prevent these risk factors or mediators may interrupt the pathway to CP and reduce CP prevalence. The trend of higher CP prevalence in low-income families warrants further investigation to reduce the CP rate in this population. Furthermore, children lived in rural areas had a higher prevalence of CP, particularly severe CP. Because people living in rural areas are less likely to use medical services [32], it is important to facilitate the access to relevant health services for children with CP residing in rural areas to help them achieving optimal development and health.

Limited evidence is available on the life expectancy of children with CP [33,34]. According to the World Health Organization Global Health Observatory data, the mortality rate of children under 5 years significantly reduced from 91 deaths per 1000 live births in 1990 to 43 deaths per 1000 live births in 2015 [35]. The mortality rate of children with severe CP in our study was 12.2−22.7 per 1000 children. Although this mortality rate is lower than the overall global mortality rate for children less than 5 years, it is higher than that in developed countries. Therefore, further studies are warranted to investigate the causes of the high mortality rate and provide improved health care to these children with CP to improve their prognosis.

The present study had some limitations. First, we used a claims-based database to investigate the prevalence of CP. The definition of CP used to classify patients was never validated in any way. We might have included children who do not in fact have CP but incorrectly get the ICD-9 codes (e.g. patient with intellectual disability, neuromuscular disorders, or genetic disorders, etc.). We may also miss children with mild CP who do not use the medical services. However, to obtain a catastrophic illness certificate to be exempted from copayment, guardians must provide relevant medical information to the National Health Insurance Bureau. Therefore, the prevalence of severe CP in this study must be very close to the true value. Second, detailed clinical information was not available in the database. Therefore, we were unable to categorize the different types of CP in the affected children. Third, the database used in the present study did not contain detailed information on family income, educational level, and ethnicity. Accordingly, we used the low income status and residential area as proxies for socioeconomic status.

## Conclusions

Our population-based study revealed that the annual prevalence of CP in children aged <7 years in Taiwan ranged from 1.9 to 2.8 per 1000 children. These CP prevalence are similar to those in Western countries. In addition, male sex, low family income, and rural residential location were associated with a higher CP prevalence. Developing a nationwide CP register system to thoroughly understand the prevalence and causes of CP and provide improved health care to children with CP is imperative.
